# Impact of prenatal environmental stress on cortical development

**DOI:** 10.3389/fncel.2015.00207

**Published:** 2015-05-27

**Authors:** Seiji Ishii, Kazue Hashimoto-Torii

**Affiliations:** ^1^Center for Neuroscience Research, Children's National Medical Center, Children's Research InstituteWashington, DC, USA; ^2^Department of Pediatrics, Pharmacology and Physiology, School of Medicine and Health Sciences, The George Washington UniversityWashington, DC, USA; ^3^Department of Neurobiology, School of Medicine, Kavli Institute for Neuroscience, Yale UniversityNew Haven, CT, USA

**Keywords:** cortical development, prenatal environmental stress, alcohol, autism, schizophrenia, maternal immune activation, gene-environment interaction, iPS cells

## Abstract

Prenatal exposure of the developing brain to various types of environmental stress increases susceptibility to neuropsychiatric disorders such as autism, attention deficit hyperactivity disorder and schizophrenia. Given that even subtle perturbations by prenatal environmental stress in the cerebral cortex impair the cognitive and memory functions, this review focuses on underlying molecular mechanisms of pathological cortical development. We especially highlight recent works that utilized animal exposure models, human specimens or/and induced Pluripotent Stem (iPS) cells to demonstrate: (1) molecular mechanisms shared by various types of environmental stressors, (2) the mechanisms by which the affected extracortical tissues indirectly impact the cortical development and function, and (3) interaction between prenatal environmental stress and the genetic predisposition of neuropsychiatric disorders. Finally, we discuss current challenges for achieving a comprehensive understanding of the role of environmentally disturbed molecular expressions in cortical maldevelopment, knowledge of which may eventually facilitate discovery of interventions for prenatal environment-linked neuropsychiatric disorders.

## Introduction

The development of the cerebral cortex consists of very intricate multifaceted steps including proliferation/differentiation of neural progenitor cells, neuronal migration and maturation (Whitford et al., 2002; Kriegstein and Noctor, 2004; Kriegstein et al., 2006; Ayala et al., 2007; Barnes and Polleux, 2009; Rakic, 2009; Rakic et al., 2009; Evsyukova et al., 2013; Lewis et al., 2013), and it can be impaired by exposure to environmental stress (Ben-Ari, 2008; Deverman and Patterson, 2009; Thompson et al., 2009). Even subtle disturbances in the development of the cerebral cortex impair cognitive and memory functions (Berger-Sweeney and Hohmann, 1997; Arnsten, 2009). Accordingly, ever increasing attention is being paid to understanding the underlying non-genomic alterations thought to govern impairment.

Alcohol is known as one of the most prevalent prenatal environmental stress, and prenatal alcohol exposure-linked impairments are categorized under the term “Fetal Alcohol Spectrum Disorder (FASD).” FASD patients show higher rates of co-morbidity with various types of neuropsychiatric problems, such as attention deficit hyperactivity disorder (ADHD) and epilepsy (Mattson and Riley, 1998). Histological analysis using postmortem tissues from FASD patients documented various anomalies in the brain, including heterotopias, microcephaly, hydrocephaly, and agenesis of the corpus callosum (Clarren and Smith, 1978; Roebuck et al., 1998; Muralidharan et al., 2013). Many of these morphological phenotypes, as well as behavioral phenotypes of human patients, have been reproduced by non-human primate, rodent and other vertebrate models of fetal alcohol exposure, and therefore, these animal models have been used for understanding etiology of FASD and other health problems linked to prenatal alcohol exposure (Miller and Nowakowski, 1991; Kelly et al., 2009; Wilson and Cudd, 2011; Patten et al., 2014). Furthermore, these animal studies found that fetal alcohol exposure particularly affects the development of the cerebral cortex, in multiple cellular events including proliferation, differentiation, apoptosis, migration, synaptogenesis and dendritogenesis, depending on the regimens and timing of exposure (Lindsley et al., 2006; Thompson et al., 2009; Miranda, 2012).

Similarly, clinical and epidemiological studies identified a variety of environmental stressors, exposure to which increases the risk of neuropsychiatric diseases (Schmitt et al., 2014). Importantly, rodent and non-human primate models of prenatal exposure to those environmental factors, including hypoxia (Golan et al., 2009; Howell and Pillai, 2014), drugs such as cocaine (Gressens et al., 1992; Cabrera-Vera et al., 2000; Stanwood et al., 2001; Lidow and Song, 2001a,b; Crandall et al., 2004; Thompson et al., 2009), and heavy metals such as methylmercury (Kakita et al., 2001; Hashimoto-Torii et al., 2014), have shown that these factors cause similar structural anomalies in the cortex as well as similar abnormal behaviors (Thompson et al., 2009). These findings imply that different environmental challenges provide common impacts on cortical development, thereby resulting in similar endophenotypes.

Here, we review recent publications that found molecular mechanisms underlying pathological cortical development elicited by exposure to prenatal environmental stress and discuss how various types of prenatal environmental stress similarly affect cortical development and increase the risk of neuropsychiatric disorders.

## Early response genes that protect or disturb cortical development under the conditions of exposure to environmental stress

Based on recent findings using prokaryotes, genes that respond (either by increase or decrease of expression) to environmental stress can be classified mainly into two groups (Mitchell et al., 2009; Levine et al., 2013; Young et al., 2013). The first group consists of genes that exhibit altered expression immediately upon exposure to multiple types of environmental insult. The second group consists of genes that exhibit altered expression profiles only upon exposure to specific types of environmental stress and are generally altered gradually post exposure. Thus, orchestrated changes in the activities of these two types of genes are likely to occur in developing cortices. The following section focuses on the first group of genes that immediately respond to environmental stress and may lead to common endophenotypes (Gluckman and Hanson, 2004), discussing how these genes change the molecular landscape of cortical development and contribute to the pathogenesis elicited by prenatal environmental stress.

### Stress responsive signaling

The cellular stress activates multiple signaling pathways that are well-positioned to help restore homeostasis upon sudden environmental changes, or, in the long run, enforce a new gene expression program so cells can tolerate the new environment. These signaling pathways and genes include molecular chaperone encoding genes, genes involved in the unfolded protein response, Mitogen-Activated Protein Kinase (MAPK) and Growth Arrest and DNA Damage 45 (GADD45) signaling pathways (Yang et al., 2009). The Heat Shock Protein (HSP) pathway is a major molecular chaperone signaling pathway, the activation of which has been identified as one of immediate molecular responses to various types of environmental stress, including alcohol, heat, heavy metals and viral infection (Nollen and Morimoto, 2002; Hashimoto-Torii et al., 2011, 2014).

**Figure 1:**
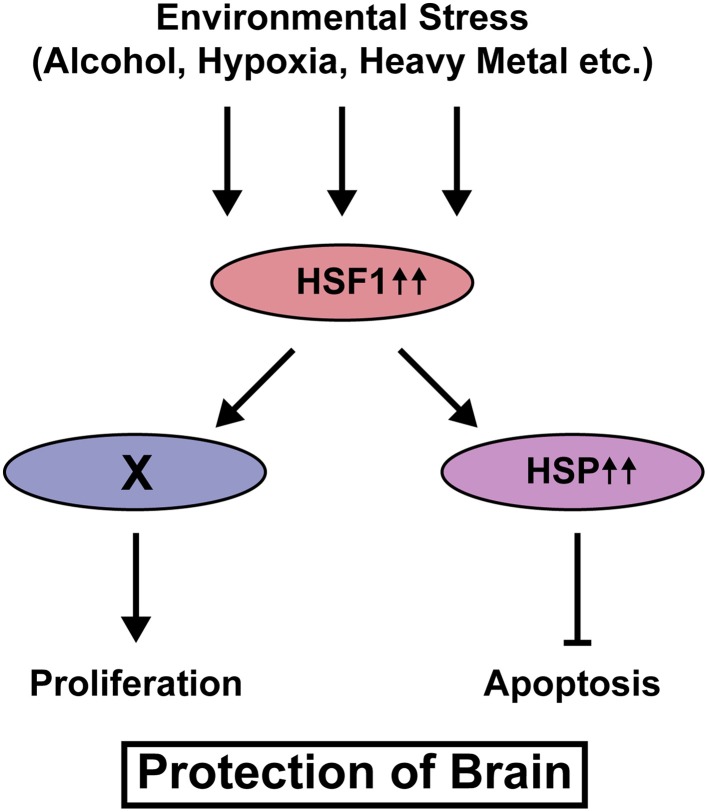
**HSF1-mediated protection of neural progenitor cells from various types of environmental stress**. Upon exposure to environmental stress, HSF1 is activated and HSPs transcribed by HSF1 inhibit cell death. HSF1 also keeps cell cycling/proliferation under stress exposure. The downstream player X is still unknown.

Our recent study using knockout mice of *Heat shock factor 1* (*Hsf1*), a canonical transcription factor that controls transcription of *Hsp* genes revealed that activation of this signaling is required to reduce the risk of cortical malformation, such as heterotopias and small size of the cortex, upon prenatal exposure to various types of environmental stress, thereby reducing susceptibility to epilepsy (Hashimoto-Torii et al., 2014). Histological analysis immediately after prenatal stress exposure revealed that the increase of these cortical malformations in *Hsf1* knockout mice is due to the increase of cell death and suspension of cell cycling, suggesting *Hsf1*'s roles in cellular protection against environmental stress. Interestingly, the canonical downstream targets of *Hsf1*, *Hsps* mediate proapoptotic effects of *Hsf1* but not the effects on cell cycling (Figure 1). El Fatimy et al. (2014) showed that, many cortical genes that are critically involved in the control of cell cycling/proliferation and the neuronal migration are under the control of *Hsf1* and the family gene *Hsf2*. Thus, the activation of HSF1 immediately alters expressions of various types of genes to protect the embryonic cortex from environmental stress.

Another example of a stress responsive transcriptional factor that protects the fetal brain from prenatal environmental stress is Nuclear Factor Erythroid 2-Related Factor 2 (Nfe2l2/Nrf2). The transcriptional activity is increased in response to such as alcohol (Narasimhan et al., 2011), kainate induced excitotoxic damage (Rojo et al., 2008a) and hydrogen peroxide induced oxidative stress (Rojo et al., 2008b). The target genes include multiple genes that encode antioxidant proteins (Dong et al., 2008; Muramatsu et al., 2013). Prenatal exposure to methamphetamine (speed) plus *Nrf2* loss of function lead to reduced motor activity, smaller body weight etc. in the offspring (Ramkissoon and Wells, 2013). Interestingly, the gender dependent differences were observed in the severity of the phenotypes.

These lines of evidence suggest that multiple cellular mechanisms provoked by the stress response genes act to ensure fetal cortical tolerance to environmental stress, and thus decrease the prevalence and severity of ensuing neuropsychiatric diseases (Hashimoto-Torii et al., 2014).

### MicroRNAs

Post-transcriptional controls have been demonstrated to be critically involved in the control of normal cortical development (Grabowski, 2011; DeBoer et al., 2013; Yano et al., 2015). MicroRNAs (miRNAs) are non-coding RNAs that are involved in post-transcriptional regulation of the expression of a wide variety of genes (Ambros, 2004). Because of their nature as short RNAs for post-transcriptional regulation of genes, they are likely to change the molecular landscape of the cell immediately and temporally in response to environmental challenges (Leung and Sharp, 2010).

In a comprehensive miRNA profiling study using a neurosphere model of alcohol exposure, Miranda and his colleagues found a reduction in expressions of *miR-21*, *miR-335*, *miR-9*, and *miR-153* 24 h after exposure (Sathyan et al., 2007).

*MiR-9* knockout mouse displays smaller brain size (Shibata et al., 2011). The analysis of those embryonic brains suggested that impaired proliferation and differentiation of neural progenitor cells in stage dependent manner may lead to the smaller brain. Consistent with this *in vivo* observation, *miR-9* knockdown inhibited the proliferation and promoted the migration of the neural progenitor cells *in vitro* (Delaloy et al., 2010). The control of these biological events by *miR-9* may be mediated by controlling expression levels of the downstream targets such as *Forkhead box G1* (*Foxg1*/*Bf1*) (Shibata et al., 2008, 2011), *embryonic lethal, abnormal vision, Drosophila like 2* (*Elavl2*/*HuB*) (Sathyan et al., 2007), *Fibroblast growth factor receptor 1* (*Fgfr1*) (Pappalardo-Carter et al., 2013), *Forkhead box P2* (*Foxp2*) (Pappalardo-Carter et al., 2013), *Stathmin 1* (*Stmn1*) (Delaloy et al., 2010), *Nuclear receptor subfamily 2, group E, member 1* (*Nr2e1*/*Tlx*) (Zhao et al., 2009; Shibata et al., 2011), *Inhibitor of DNA binding 4* (*Id4*) (Shibata et al., 2008), *Paired box 6* (*Pax6*) (Shibata et al., 2011), *Meis homeobox 2* (*Meis2*) (Shibata et al., 2011), *GS homeobox 2* (*Gsh2*) (Shibata et al., 2011), *Islet1* (*Isl1*) (Shibata et al., 2011), *RE1-silencing transcription factor* (*Rest*) (Packer et al., 2008), and *Actin-like 6A* (*Actl6a*/*BAF53a*) (Yoo et al., 2009). Thus, reduced expression of *miR-9* by alcohol exposure is also likely to inhibit those events by the similar mechanism. The miR-153 and miR-21 also similarly control the cellular proliferation (Zhong et al., 2012; Wu et al., 2013).

Reduction of *miR-9* expression and the target gene expressions in the zebrafish whole-embryo (Tal et al., 2012) and the embryonic forebrain (Pappalardo-Carter et al., 2013) exposed to alcohol also supports this hypothesis. However, in the conditions of exposure to different contexts of maternal stress induced by such as restraint of the body and forced swimming, expression of *miR-9* was increased in the brain of offspring (Zucchi et al., 2013). Similarly, the expression of *miR-21* has also been reported to be increased in the different ambience, such as in the mouse brain exposed to ionizing radiation (Shi et al., 2012a), in the endothelial cells under the exposure to shear stress (Weber et al., 2010), and in the embryonic fibroblasts exposed to arsenite (Ling et al., 2012). The expression of *miR-153* is also upregulated by hydrogen peroxidase induced oxidative stress (Narasimhan et al., 2014) and nicotine exposure (Tsai et al., 2014). These lines of evidence indicate that the microRNAs are susceptible to the environmental changes and that the overall changes of various types of microRNAs may determine the phenotypes specific to types/regimens of the environmental stress exposure. The fact that miR-335 knockdown reverses the effects of miR-21 knockdown in the cell proliferation and death also supports this possibility (Sathyan et al., 2007).

## Maternal, placental, and extracortical tissues exhibit indirect effects as a result of environmental stress

Beside direct molecular changes within embryonic cortical cells, evidences exist that indirect impacts of environmental stress from maternal, placental, and other extracortical tissues exert a critical influence on cortical development (Velasquez et al., 2013).

Maternal infection is well defined by epidemiological studies as a risk factor for neurodevelopmental disorders such as autism and schizophrenia (Hagberg et al., 2012; Depino, 2013; Meldrum et al., 2013). Mouse offspring that have been exposed to maternal infection display abnormalities reminiscent of the behavioral, histological, and molecular characteristics of autism (Patterson, 2011), while fetal brain infection does not cause these abnormalities (Meldrum et al., 2013). Mouse offspring exposed to maternal immune activation (MIA), which is elicited by poly-riboinosinic-polyribocytidylic acid or lipopolysaccharide, also reproduce the behavioral and histological abnormalities of autism (Meyer et al., 2006; Smith et al., 2007; Hsiao et al., 2012; Carpentier et al., 2013), suggesting that activation of maternal immune system triggered by infection is critical for manifestation of deficits. These early findings have proven MIA model useful in the investigation of the molecular mechanisms at play in unraveling maternal effects on the pathophysiology of autism.

Smith et al. (2007) demonstrated that a proinflammatory cytokine interleukin-6 (IL-6) supplied from the maternal tissues might mediate the MIA effects on the fetal cortex. A single maternal injection of IL-6 in the middle of corticogenesis causes deficits in prepulse inhibition and lateral inhibition in the offspring (Smith et al., 2007), both of which are linked to autism and schizophrenia (Solomon et al., 1981; Wynn et al., 2004; Bertone et al., 2005; Perry et al., 2007). They also demonstrated that inhibition of IL-6 by application of the antibody or using the knockout dam, significantly ameliorated such as cognitive and exploratory deficits in mouse offspring exposed to MIA (Smith et al., 2007). The gene expression profiles were also reversed by inhibition of IL-6 in the cortices of the MIA offspring. These results provided evidence that IL-6 may owe the indirect effects of MIA on fetal cortical development.

Indirect effects of MIA on cortical development may also involve the effects from gastrointestinal tissues of offspring. Autism is often associated with gastrointestinal barrier defects (Buie et al., 2010; Coury et al., 2012), and rodent MIA models reproduce these defects (Hsiao et al., 2013). Hsiao and colleagues made an interesting observation that probiotic treatment of gastrointestinal barrier defects improved behavioral abnormalities such as anxiety-like behavior, decreased prepulse inhibition, and deficits in ultrasonic vocal communication in the MIA offspring. Their study also suggested the possibility that gastrointestinal barrier deficit-induced increase of serum metabolites such as 4-ethylphenylsulfate, indolepyruvate, glycolate, imidazole propionate, and N-acetylserine, may contribute to behavior abnormality in the MIA offspring (Hsiao et al., 2013). Of these, the most dramatically affected metabolite, 4-ethylphenylsulfate, has been known as a uremic toxin, and the administration of this metabolite induces anxiety-like behavior in the mouse (Hsiao et al., 2013). As a recent study suggested the link between the uremic toxin and the depression in the chronic kidney disease (Hsu et al., 2013), the 4-ethylphenylsulfate in serum may be the common factor that affects the brain function in various pathophysiological conditions.

Serotonin derived from placenta may also indirectly affect embryonic brain development. Recent studies demonstrated that the placenta is the major source of serotonin at early embryonic stage, while the dorsal raphe nuclei in the hindbrain take over from late embryonic stage to adulthood (Bonnin et al., 2011). Abnormal serotonin levels in the brain have been linked to autism (Chugani et al., 1999; Whitaker-Azmitia, 2001; Gaspar et al., 2003), and the role of serotonin in the normal development of thalamocortical projections also has been reported (Bonnin et al., 2007). In addition, it has been demonstrated that prenatal intake of selective serotonin reuptake inhibitors increases the risk of cognitive impairment in mouse progeny (Smit-Rigter et al., 2012; Kinast et al., 2013). Importantly, serotonin level is lower in the cortices of the offspring exposed to environmental stress such as maternal infection (Fatemi et al., 2008; Wang et al., 2009) and cocaine (Cabrera-Vera et al., 2000). Therefore, environmental stressors may indirectly affect the cortical development as a result of disruption in the synthesis/release of serotonin in/from the placenta (Velasquez et al., 2013).

### Interaction between a susceptible genotype and environmental risk factors

Genome wide association studies have shown a polygenic component contributes to the risk of schizophrenia and autism (Purcell et al., 2014). Similarly, many epidemiological studies as well as the aforementioned results from studies of animal exposure models have shown these disorders also include a “polyepigenetic” component that is influenced by various types of environmental stress (Weinberger, 1987; Caspi and Moffitt, 2006; Ben-Ari, 2008; van Os et al., 2010; Bregant et al., 2013). However, just how the polyepigenetic component increases the risk of disease manifestation by interacting with polygenic component is largely unknown.

**Figure 2:**
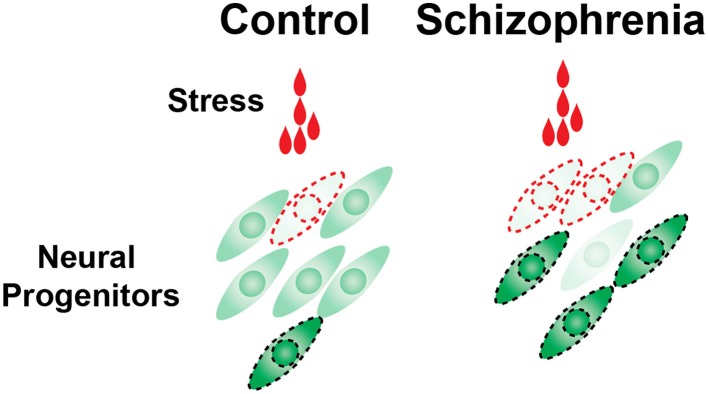
**Cell-to-cell variability of HSF1 activation in response to environmental challenges is increased in schizophrenia neural progenitor cells**. The number of cells that are in the levels of excess or very little activation of HSF1 was increased in the schizophrenia cells (Hashimoto-Torii et al., 2014; Brennand et al., 2015). These outlier cells may be at the risk of manifesting pathophysiological features (indicated by the cells surrounded by broken lines).

One relatively new approach to help answering this question is the use of induced Pluripotent Stem (iPS) cells taken from subjects diagnosed with polygenic diseases such as schizophrenia or autism. iPS cells are not only becoming useful tools to obtain functional human cortical neurons (Mariani et al., 2012; Shi et al., 2012b; Espuny-Camacho et al., 2013; Lancaster et al., 2013) for understanding the pathogenesis of disease, but are also being utilized for drug screening (Han et al., 2011). To examine potential interactions between genetic predisposition and the environmental risk factors, we recently used iPS cells derived from schizophrenia patients, and exposed the differentiated neural progenitor cells to environmental stress including alcohol, methylmercury and hydrogen peroxide. Single cell RNA detection revealed augmented cell-to-cell variable activation of HSF1-HSP signaling in the schizophrenia patients' neural progenitor cells, individual cell lines of which carry different genetic risks for schizophrenia (Figure 2). This finding suggests that variable responses of HSF1-HSP signaling among a population of neural progenitor cells exposed to environmental stress is predetermined by genetic predisposition and may increase the risk of the onset of schizophrenia as well as other neuropsychiatric diseases (Hashimoto-Torii et al., 2014; Brennand et al., 2015).

Using *Disrupted-in-schizophrenia-1* gene (*Disc1*) mutant mice combined with MIA, *in vivo* evidence for the interaction of gene and prenatal environment in the pathogenesis of schizophrenia and depression was also provided. The *Disc1* is one of the risk genes for psychiatric disorders such as schizophrenia and mood disorders (St Clair et al., 1990; Millar et al., 2000). The transgenic mice expressing the dominant negative form of *Disc1* that was found in the patient (Millar et al., 2000), displayed hyperactivity and impaired social interaction (Pletnikov et al., 2008). When this transgenic mouse was subjected to MIA, neurobehavioral phenotypes such as anxiety, depression-like behavior, and a decrease in social interaction and an increase in aggressiveness were unraveled (Abazyan et al., 2010). Two other *Disc1* mutant mouse lines with point mutations at Q31L and L100P, which show schizophrenia and depression related phenotypes, respectively (Clapcote et al., 2007), were also subjected to MIA. MIA exposure augmented the impairment in prepulse inhibition, lateral inhibition, spatial object recognition, and social motivation of those *Disc1* mutant mice (Lipina et al., 2013). Importantly, the production of IL-6 was concomitantly increased by the combination of *Disc1* mutations and the MIA in the fetal mouse brains (Lipina et al., 2013). Thus, these mouse models that combine *Disc1* mutation and MIA will become powerful models for understanding the molecular mechanisms underlying interactions between the gene and prenatal environmental factors that increase the risk of the psychiatric diseases.

## Outlook

As outlined in this review, research on polyepigenetic mechanisms associated with many types of environmental stress that disturb cortical development and on potential prophylactic or preventative interventions of these disturbances are just beginning to emerge. To further facilitate this type of research, patient-derived iPS cells will become one of several powerful tools. Although there are a number of limitations in their use, easy application of environmental stress and the potential for high throughput analysis substantiate their usefulness. Challenges include: (1) limited availability of iPS cell lines that are fully characterized; (2) lack of validated differentiation protocols for specific types of neurons; and (3) lack of validated *in vivo* approaches (e.g., efficient transplantation methods to animal models, etc.) that allow observation of the iPS cells during cortical development.

A type of the environmental stress can lead to various phenotypes in the cerebral cortex, however, this variability cannot be explained exclusively by different regimens of exposure. Recent studies have revealed potential factors that may affect the resultant phenotypes, including gender (Mooney and Varlinskaya, 2011; Ramkissoon and Wells, 2013) and probabilistic molecular responses of individual cells to the environmental stress (Hashimoto-Torii et al., 2014) (Figure 2) etc. Thus, the next important questions will be: (1) if such molecular differences of individual cells elicited by environmental stress are sustained for long periods of time and ultimately result in altered cortical function, and (2) which molecules mediate the gender specific effects of prenatal environmental stress.

Another recent interesting observation that needs to be addressed at the molecular level is the transgenerational effects of prenatal exposure to environmental stress, as reported in the cases of alcohol (Govorko et al., 2012). This observation opens up a whole new field of research that might eventually lead to an understanding of why FASD and other environment-linked disorders show familial and geographical linkages.

### Conflict of interest statement

The authors declare that the research was conducted in the absence of any commercial or financial relationships that could be construed as a potential conflict of interest.

## Abbreviations

iPS cells: induced Pluripotent Stem cells
FASD: fetal alcohol spectrum disorder
HSP(s): Heat Shock Protein(s)
HSF1: Heat Shock Factor 1
MIA: maternal immune activation
IL-6: interleukin-6
DISC1: disrupted-in-schizophrenia-1.
